# The impact of healthcare costs in the last year of life and in all life years gained on the cost-effectiveness of cancer screening

**DOI:** 10.1038/sj.bjc.6605018

**Published:** 2009-04-14

**Authors:** I M C M de Kok, J J Polder, J D F Habbema, L-M Berkers, W J Meerding, M Rebolj, M van Ballegooijen

**Affiliations:** 1 Department of Public Health, Erasmus MC, University Medical Center Rotterdam, Rotterdam, The Netherlands; 2 TRANZO Department, Tilburg University, Tilburg, The Netherlands; 3 National Institute for Public Health and the Environment, Centre for Public Health Forecasting, Bilthoven, The Netherlands

**Keywords:** cost-effectiveness, economic evaluation, screening

## Abstract

It is under debate whether healthcare costs related to death and in life years gained (LysG) due to life saving interventions should be included in economic evaluations. We estimated the impact of including these costs on cost-effectiveness of cancer screening. We obtained health insurance, home care, nursing homes, and mortality data for 2.1 million inhabitants in the Netherlands in 1998–1999. Costs related to death were approximated by the healthcare costs in the last year of life (LastYL), by cause and age of death. Costs in LYsG were estimated by calculating the healthcare costs in any life year. We calculated the change in cost-effectiveness ratios (CERs) if unrelated healthcare costs in the LastYL or in LYsG would be included. Costs in the LastYL were on average 33% higher for persons dying from cancer than from any cause. Including costs in LysG increased the CER by €4040 in women, and by €4100 in men. Of these, €660 in women, and €890 in men, were costs in the LastYL. Including unrelated healthcare costs in the LastYL or in LYsG will change the comparative cost-effectiveness of healthcare programmes. The CERs of cancer screening programmes will clearly increase, with approximately €4000. However, because of the favourable CER's, including unrelated healthcare costs will in general have limited policy implications.

Cancer screening induces both costs and savings (Stone *et al*, 2000). Savings occur due to avoided treatment of advanced disease and palliative care, whereas the costs increase because of screening activities and because more cases of (preinvasive) neoplasia will be found than in the situation without screening.

Cost-effectiveness analysis, the standard analytic tool supporting medical decision making, involves estimating the costs and effects of an intervention compared with an alternative, for example, the care that would be given if the intervention was not used at all, or with a different intensity of the intervention under investigation, such as less frequent screening (Russell *et al*, 1996). One of the most persistent unresolved issues in the use of cost-effectiveness analysis is the application of the healthcare costs related to postponed death, or more in general, of unrelated healthcare costs in life years gained (LysG; Garber *et al*, 1996). It is increasingly argued that economic evaluations should include these costs to be consistent (Nyman, 2004), as LysG due to spending unrelated healthcare costs are generally included. However, the most common practice is to include medical cost for illnesses related to the intervention, and to ignore increases in medical expenditures due to other illnesses that arise during the LysG. A particular kind of unrelated healthcare costs in LysG are medical costs related to death from another cause (Meltzer, 1997). As increasing life expectancy due to preventive interventions simply postpones the costs related to death, it is believed that not considering these costs would overestimate the savings in healthcare costs (Gandjour and Lauterbach, 2005). Future unrelated healthcare costs might be large enough to raise the CER to such a degree that the ranking of alternative interventions can be changed, which constitutes important information to policymakers. The impact is greatest when the intervention primarily extends life, such as is the case with cancer screening (Garber and Phelps, 1997).

We considered both: (1) including the healthcare costs related to postponed death, regardless of its cause and (2) more in general of including the unrelated healthcare costs in LysG. As a proxy of costs related to death, we studied the healthcare costs in the last year of life (LastYL), discerning between deaths caused by a specific cancer (focusing on cancers for which screening is recommended or at least under discussion) from deaths due to any cause. We also investigated the healthcare costs in the LysG and how they depend on age. Finally, to understand the potential impact, we calculated the increase these costs would make on the estimated CER.

## Materials and methods

We obtained health insurance data for 1998 and 1999 for a sample of 2.1 million inhabitants, representing 13.4% of the whole Dutch population in 1999. The study group is representative for the Dutch population regarding age, gender, and cause of death (Polder *et al*, 2006). Of the study population, 66.5% was insured by social health insurance and 33.5% by private health insurance, which was in line with the corresponding distribution in the general population in 1999 (63.5 *vs* 36.5% insured by social and private health insurance, respectively) (Statistics Netherlands, 2008). Both insurance schemes covered a similar package of healthcare services. The health services we included were the expenses for physicians, primary care, hospitals, drugs, and related services. We also included expenditures on nursing homes and home care. A detailed description of the health services included, the study group and the cost calculation, and projection is presented elsewhere (Polder *et al*, 2006).

The data on nursing homes and home care were linked with health insurance data at individual level, using birth date, sex, and zip code. The registration of home care was complete. The nursing homes registry covered 65% of the users of nursing home care. As the coverage appeared to be non-selective, we adjusted the average nursing home costs in our study population, using a correction factor depending on coverage per geographical area (on average: 100/65).

Subsequently, to obtain date and cause of death, the data of the health insurance companies was linked with mortality data from Statistics Netherlands, using birth date, sex, and zip code. In the final analysis, we distinguished between costs of survivors (*n*=2 093 748) and decedents (*n*=14 839), the latter stratified by cause of death. All individuals who entered or dropped out the study population in 1998 or 1999, for example due to a change in their insurance scheme, or individuals who died in 1998, were excluded from the study. Also, individuals who died in 2000 were excluded, because part of their health expenditures in 1999 could include costs related to death.

### Healthcare cost in the LastYL

The costs before dying were approximated by the healthcare costs in the LastYL. For decedents in 1999, we calculated health expenditures in the 365 days before death. For privately insured decedents, we had information on the date of death and total healthcare expenditure for the individual years 1998 and 1999, as well as the exact health expenditures during the LastYL. For individuals with a social health insurance scheme, we had information on the date of death and the total healthcare expenditure for the separate years 1998 and 1999, but not the exact health expenditures during the LastYL. We, therefore, interpolated the cost in the LastYL for individuals with social health insurance. As health expenditure is increasing within the LastYL, we used a non-linear interpolation method. We divided the privately insured population into 12 groups according to the month of death in 1999, and for each group calculated the fraction of the 1998 expenditure that belonged to the LastYL. As the month of death of the decedents with social insurance schemes was known, we could use these fractions to estimate the share of their individual 1998 expenditure that belonged to the LastYL. We calculated the costs in the LastYL under the assumption that the distribution of the costs over the LastYL (not the level of those costs) is comparable for both groups of insurance.

**Figure 1:**
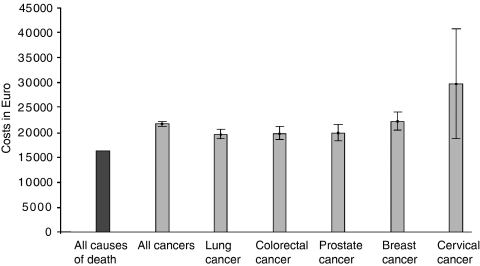
Mean (95% confidence intervals) healthcare costs in the last year of life, by cause of death (excluding nursing home care).

**Figure 2:**
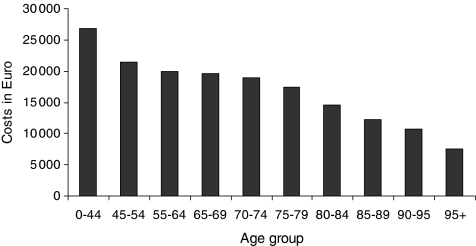
Mean healthcare costs in the last year of life by age group, all causes of death.

We calculated the 95% confidence intervals for healthcare costs in the LastYL on the basis of a lognormal distribution. This was not possible for the costs of nursing homes, because for this cost category, only average figures on group level were available (by age, gender, and cause of death). The costs before dying were stratified by age, gender, and cause of death. We stratified the causes of death into five types of cancer that are potentially preventable due to screening (lung, colon, prostate, breast, and cervical cancer) all cancer and causes (Figure 1). On account of the small number of decedents in the younger age groups, we used an asymmetric age structure: 0–44, 45–54, and 55–64 years, and from then on 5-year categories ending with the category 95 years and older (Figure 2).

### Healthcare costs in LysG

**Figure 3:**
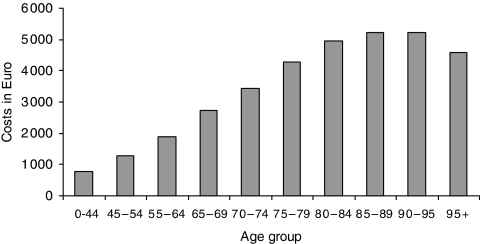
Mean healthcare costs per life year, by age group.

We calculated the average healthcare costs in the LysG, by gender and age category at which death is prevented. To do so, we first calculated the average annual healthcare costs by age and gender, using costs in ‘survivors’ (Figure 3). In this way, these annual costs were cleared from the costs in the LastYL. Second, we summated these annual healthcare costs for each specific expected life year gained (LyG). The expected number of LysG for individuals whose death is prevented (postponed) by screening were estimated by age at which death is prevented and gender, using life tables for the Dutch population (Statistics Netherlands, 2007). Third, we added up the costs in the expected LysG in survivors and the costs in the LastYL.

### Increase in costs per LyG

We calculated the increase in costs per LyG when taking into account both the unrelated healthcare costs of dying (Table 1) and the unrelated healthcare costs during LysG (Table 2), by gender and age at which death is prevented (postponed), undiscounted and discounted (costs and LysG by 3% towards the age of 50 years, assuming screening starts at that age). To do so, we divided the calculated healthcare costs in the substituting LastYL and the healthcare costs in LysG, respectively, by the average number of LysG.

As an example, we calculated the impact of the above estimated extra costs per LyG on the CER of breast cancer screening, assuming that the mean age at breast cancer death (68 years (Netherlands Cancer Registry (NCR), 2005)) is the average age at which death is prevented by breast cancer screening in our population. Here, we used evidence from literature on cost-effectiveness of breast cancer screening without accounting for the evaluated unrelated healthcare costs (Groenewoud *et al*, 2004). Included in this cost-effectiveness analysis were costs of screening, and the costs of diagnostics, primary treatment, follow-up, and palliative care of breast cancer. The effect of screening was estimated by the difference in number of life years lost due to breast cancer with and without screening. Costs and effects were adjusted with a 3% annual discount rate.

All costs and CERs were indexed according to the price level of 2008 using the consumer price index.

## Results

The healthcare costs in the LastYL were significantly higher for individuals who died from cancer than for those who died from any cause (€21 700 *vs* €16 300; Figure 1). The costs in individuals who died of cervical cancer were the highest (€29 700), partly explained by the fact that cervical cancer patients die at a relative younger age. Nevertheless, the difference with dying from other cancers was not significant due to the small number of cervical cancer death cases. Considering all causes of death, healthcare costs in the LastYL decreased with age (€26 800 in age-group 0–44 to €7500 in age-group 95+; Figure 2). The average yearly healthcare costs increased with age (€800 in age group 0–44 years to €4600 in age-group 95+ years; Figure 3).

Table 1 shows the increase in costs per LyG if unrelated healthcare costs in the postponed LastYL are taken into account. After discounting, costs per LyG increase for men on average by €680, and for women by €480. This increase in costs per LyG increases with age at which death is prevented, despite the fact that the costs in the LastYL decrease with age (Figure 2). The reason is that the number of LysG, the denominator in the equation, decrease with age.

Table 2 shows the increase in costs per LyG when unrelated healthcare costs in LysG is taken into account. After discounting, costs per LyG increase for men on average by €4100 and for women by €4000. This increase becomes larger with age at which death is prevented, because the yearly healthcare costs increase with age (Figure 3).

The effect of discounting on the increase in costs per LyG is limited, because both costs, as well as LysG, are discounted at the same rate (3% per year).

The overall impact depends on the CER before the adjustment. For breast cancer screening in the Netherlands, for example, this ratio was estimated at approximately €2700 per LyG (UK£1515 in 2002; Groenewoud *et al*, 2004). If healthcare costs unrelated to the prevented breast cancer in the LysG are taken into account, the CER would increase to approximately €7300 per LyG, which means an increase of 171%. If only the unrelated healthcare costs in the postponed LastYL are taken into account, the CER would increase to approximately €3200 per LyG, which is an increase of 20%. The effect would have been smaller if screening prevented death at younger ages, whereas a larger increase would occur if age at prevented death would have been higher.

## Discussion

We showed that the healthcare costs in the LastYL are higher than the mean yearly healthcare costs and that the former costs decrease with age, whereas the latter increase with age. The costs in the LastYL are higher for individuals who die from cancer than for those who die from any cause. If we take medical costs in the postponed LastYL into account, costs per LyG increase between €360 and €890, depending on age and gender. These higher costs occur because in traditional cost-effectiveness calculations the savings of prevented cure and care are overestimated, as the costs of dying are only postponed rather than avoided. If, instead, we take unrelated healthcare costs in all LysG into account, the costs per LysG increase between €3100 and €5200. This effect increases with age at which death is prevented by screening.

The specification of which costs to include in the analysis depends on the perspective of the economic evaluation (Russell *et al*, 1996). Unrelated healthcare costs in LysG need to be included from the perspective of the health services. From the societal perspective, however, all costs and effects resulted from the intervention need to be considered. Therefore, if medical costs in LysG are included in the evaluation, non-medical costs in LysG and productivity gains should also be included. However, because of practical concerns, for example, lack of data, and unresolved theoretical issues surrounding the inclusion of costs in added life years, researchers often do not include these costs. Thereby, researchers can choose to neglect unrelated healthcare costs in LysG, as the guidelines for economic evaluations are not explicit on the inclusion of these costs. As costs related to death can be regarded as a correction of the savings, because they are not prevented, but only postponed by prevention (Gandjour and Lauterbach, 2005), they can be included from the perspective of the health services as well as from the societal perspective.

The impact of including the unrelated healthcare costs in the LastYL was limited, because prevention of cancer death on average replaces a rather expensive LastYL of relatively young individuals with a less costly LastYL at a higher age. The fact that the LastYL of individuals who die of cancer is relatively expensive was also found by studies from the United States (Riley *et al*, 1987; Riley and Lubitz, 1989; Koroukian *et al*, 2006) and from Australia (Kardamanidis *et al*, 2007). That unrelated healthcare costs in the LastYL are higher for cancer patients than for other decedents can be explained by intensive and more expensive treatment. Furthermore, the group of other decedents includes a substantial fraction of ‘sudden death’, among others by myocardial infarctions, strokes, and accidents (Wong *et al*, 2006). Given the higher costs of cancer deaths and because healthcare costs related to death decrease with age, simply shifting the costs related to a prevented cancer death to the future will underestimate the total costs of the intervention. Discounting of future costs reduces the impact on the CER of including the postponed costs even further. On the other hand, if age-related disutility during added life years is considered, the impact on the CER (costs per quality-adjusted LyG) would increase, as added life years are lived at older ages, and the quality of these life years is relatively less.

As the CER of cancer screening increases when unrelated healthcare costs in the postponed LastYL or in all LysG are taken into account, including these costs in the cost-effectiveness analyses may have impact on the policy decisions about a particular screening programme, also because the optimal (from a cost-effective point of view) screen policy may change. On the other hand, if these costs are included, for example, for the current breast cancer screening programme in the Netherlands, the costs per LyG still are acceptable according the Dutch threshold value of about €20 000 per quality-adjusted LyG. The CER (without including costs in the LastYL or in LysG) of other cancer screening programmes in the Netherlands, such as cervical cancer screening (€9500 per LyG (van Ballegooijen *et al*, 2006)), and faecal occult blood test-based colorectal cancer screening (€15 000 per LyG (Pignone *et al*, 2002)), also remain under the acceptability threshold. The increase in CER for colorectal cancer screening will be higher than the increase in CER for cervical cancer screening, because the average age at which death is prevented is higher for colorectal cancer screening than for cervical cancer screening (Tables 1 and 2).

By including unrelated healthcare costs in the LastYL as well as in LysG, the CER of prevention of lethal illnesses can become less favourable compared with the prevention of non-lethal illnesses. Vaccination during childhood against, for example, mumps and rubella, prevents a lot of severe disabilities, but in developed countries, individuals rarely die of those diseases in the absence of vaccination. As a consequence, there are no healthcare costs related to postponed death or in LysG in this situation. Therefore, the CER of prevention of these non-lethal illnesses will not change.

Bonneux *et al* (1998) showed that elimination of fatal diseases, such as cancer, significantly increases the life-time expected healthcare costs. They found that the elimination of cancer will increase the healthcare costs with €2300 (£912 in 1988) and €3000 (£1190 in 1988) per LyG for men and women, respectively. The difference between these results and our estimated increase in costs per LyG, when taking into account healthcare costs in LysG, is explained by the fact that healthcare costs have increased in the last 20 years (World Health Organisation (WHO), 16/02/2009).

In the meantime, when cost-effectiveness analyses are used for policy decisions, it is important that any type of costs are either consistently excluded or consistently included (van Baal *et al*, 2007).

### Strengths and weaknesses

The strength of our study is that we linked healthcare costs to the official mortality register on the individual level, and were, therefore, able to analyse cost differences per cause of death. Also, we included all medical care costs, whereas most other studies focus on hospital care only. Thereby, due to the fact that the government heavily regulates the Dutch healthcare system, the claims on the health insurance are very comparable to the actual healthcare costs that makes the estimation of the medical costs reliable.

A potential weakness is that we used average annual healthcare expenditures as an estimate of the yearly healthcare costs of individuals whose cancer death is prevented by cancer screening, whereas individuals whose cancer death is prevented may tend to have a more active healthcare-seeking pattern. Also, there is some evidence that individuals diagnosed with cancer have an increased risk of other diseases due to the fact that the risk factor that played a role in the development of cancer can cause other diseases as well (Eakin *et al*, 2007; Soerjomataram, 2007). The symptoms or illnesses that generate the possible higher expenses in individuals whose cancer death is prevented are not directly related to the illness for which death was prevented, and are therefore considered as unrelated healthcare costs. As a consequence, the assumption that these individuals have average annual unrelated healthcare expenditures, may have led to a slight underestimation of the increase in costs per LyG.

Finally, as we mentioned before, it is argued that for an evaluation from a societal perspective non-medical costs and benefits in LysG, that is, productivity gains, paid pensions, and non-health-related quality of life need to be included in economic evaluations (Liljas *et al*, 2008). We did not take into account these issues, as they go beyond the scope of this study, but should be studied in the continuing debate on costs and benefits of prevention.

In conclusion, taking the unrelated healthcare costs in the postponed LastYL or during all LysG into account in cost-effectiveness analyses will change the relative cost-effectiveness of healthcare programmes and may influence priority settings. The CERs of cancer screening programmes will clearly increase, with approximately €4000. However, because of the favourable CERs, including unrelated healthcare costs in LysG will, in general, have limited policy implications.

## Figures and Tables

**Table 1:** Increase in costs per life year gained when taking into account healthcare costs in the postponed last year of life, by sex and age at which death is prevented (discounted at 0 and 3%)

**Age at prevented**	**0% discounting**		**3% discounting**	
**death (years)**	**Men**	**Women**	**Men**	**Women**
50–54	€ 600	€ 460	€ 500	€ 360
55–59	€ 710	€ 540	€ 630	€ 450
60–64	€ 880	€ 650	€ 700	€ 500
65–69	€ 1,100	€ 790	€ 800	€ 550
70–74	€ 1,360	€ 980	€ 890	€ 610
Mean	€ 890	€ 660	€ 680	€ 480

**Table 2:** Increase in costs per life year gained when taking into account unrelated healthcare costs in life years gained, by sex and age at which death is prevented (discounted at 0 and 3%)

**Age at prevented**	**0% discounting**		**3% discounting**	
**death (years)**	**Men**	**Women**	**Men**	**Women**
50–54	€ 3280	€ 3370	€ 3120	€ 3190
55–59	€ 3650	€ 3710	€ 4150	€ 3880
60–64	€ 4140	€ 4140	€ 4040	€ 4400
65–69	€ 4750	€ 4640	€ 4640	€ 4580
70–74	€ 5370	€ 5150	€ 5170	€ 5070
Mean	€ 4110	€ 4100	€ 4100	€ 4040
